# The growing burden of mental health and the role of digital technologies in the Global South: policies, cultural contexts, and community-based transformations—a systematic review of gray literature evidence

**DOI:** 10.3389/fpubh.2026.1875110

**Published:** 2026-07-21

**Authors:** Joseph Odhiambo Onyango

**Affiliations:** Centre for Social Behavior Change and Strategic Foresights, Strathmore Business School, Strathmore University, Nairobi, Kenya

**Keywords:** Global South mental health, digital mental health, grey literature, LMIC, cultural values, mental health burden, policy, community-based transformations

## Abstract

**Background:**

Mental health conditions are among the fastest growing and most inequitably distributed global disease burdens. Structural determinants- poverty, conflict, climate change, and the COVID-19 pandemic compound a persistent mismatch between internationally developed policy frameworks and the cultural and infrastructural realities of populations in the Global South.

**Objective:**

This systematic review identified, appraised, and synthesized gray literature evidence on (1) the growing mental health burden across Global South regions (2015–2025); (2) digital technology responses in Global South LMIC contexts; and (3) policy, cultural, and community-based factors shaping mental health transformation efforts.

**Methods:**

A pre-registered systematic gray literature review spanned five source tiers. Following systematic screening and AACODS quality appraisal, 140 records published between 2015 and 2025 were included.

**Results:**

Four themes emerged: (1) an accelerating, structurally driven mental health burden across Global South regions; (2) a severe and entrenched treatment gap sustained by chronic underinvestment, workforce shortages, and stigma; (3) a rapidly expanding but uneven digital mental health landscape, in which SMS-based and CHW-mediated platforms achieve substantially greater reach than smartphone-dependent tools; and (4) significant structural, cultural, and policy barriers constraining scale-up. The effectiveness of digital mental health interventions (DMHIs) varies by modality, population, and setting, and is not a single generalizable finding.

**Conclusion:**

The mental health burden in the Global South is growing faster than existing systems can address. Transformative progress requires sustained investment in CHW-mediated digital tools, culturally co-designed platforms, decolonized research partnerships, and mental health policies that center Global South communities as co-producers of their own mental health futures.

## 1 Introduction

Mental health conditions are among the most significant, fastest growing, and most inequitably distributed contributors to the global burden of disease. The World Health Organization's (WHO) World Mental Health Report (1, 46, 60) estimated that approximately one billion people worldwide live with a mental health condition, with depressive and anxiety disorders alone accounting for over 970 million prevalent cases. The Global Burden of Disease Study 2021 documented that the age-standardized prevalence of mental disorders increased by 4.74% per year between 2019 and 2021, driven largely by the COVID-19 pandemic, and projected continued increases, particularly among those under 24 years of age, through 2050 (2). The economic costs are equally alarming: untreated mental illness costs the global economy over USD 1 trillion annually in lost productivity, a figure projected to escalate to USD 6 trillion by 2030 without decisive action (3).

This burden falls most heavily on the Global South—low- and middle-income countries (LMICs) in sub-Saharan Africa, South and Southeast Asia, Latin America and the Caribbean, and the Middle East and North Africa—which account for approximately 81% of global mental health disability-adjusted life years (DALYs) yet receive less than 10% of global mental health resources (1). The mental health treatment gap, the proportion of people with mental health conditions receiving no care, exceeds 75% across most of Latin America and the Caribbean (4), surpasses 85% in many Asian LMICs, and can exceed 90% for common mental disorders in parts of sub-Saharan Africa (5). In the African region alone, an estimated 10% of the population lives with a mental disorder, yet fewer than one in 10 receives any treatment (6). These figures represent not simply a health crisis but a human rights emergency with profound developmental consequences.

The growing burden in the Global South is driven by a convergence of structural determinants. Poverty and economic insecurity are among the most robust predictors of the prevalence of mental disorders (7). Armed conflict and forced displacement—geographically concentrated in LMICs—generate significant and enduring mental health sequelae, including post-traumatic stress disorder (PTSD), depression, and anxiety (8, 51). Climate change and natural hazards disproportionately affect developing countries while generating significant mental health burdens (9). The COVID-19 pandemic created an estimated 28% increase in major depressive disorders and a 26% increase in anxiety disorders globally in 2020 alone, with the greatest impact in countries with the weakest pre-existing mental health systems (10, 47, 51).

Beyond epidemiological factors, the treatment gap in the Global South is compounded by a persistent mismatch between internationally developed mental health policy frameworks and the cultural realities of the communities they are designed to serve. The WHO's Comprehensive Mental Health Action Plan (CMHAP) and the mhGAP Intervention Guide are the primary global templates for mental health system development in LMICs (11). These frameworks have generated real momentum in policy adoption, yet gray literature from national ministries of health and regional health organizations consistently documents that their implementation is constrained by the dominance of biomedical classification systems that do not map onto local idioms of distress (12), the marginalization of traditional healing systems and faith-based networks that serve as primary mental health resources for the majority of affected populations (13), and a fundamental epistemological asymmetry in which Global North frameworks are exported to Global South contexts rather than co-produced with them (14).

Digital technologies have emerged as a potentially transformative response to the mental health treatment gap. Mobile phone penetration in sub- Saharan Africa exceeds 50% of the population, and although uneven, wireless connectivity is expanding rapidly across South Asia and Latin America (15). Digital mental health interventions (DMHIs), encompassing mHealth applications, Short Message Service (SMS) and voice platforms, web-based therapy, telehealth, artificial intelligence (AI)-assisted chatbots, and peer support platforms, have demonstrated feasibility and preliminary effectiveness across diverse LMIC contexts (16, 17). These modalities are not interchangeable: they differ markedly in infrastructure dependence, cost, literacy requirements, and degree of human mediation. The evidence base for “effectiveness” is therefore not a single, generalizable question but a series of context- specific ones that must be examined separately for each modality, population, and setting—a distinction this review makes explicit in its synthesis (Section 3.3.3). International organizations, including the World Health Organization (WHO), the United Nations Children's Fund (UNICEF), the World Bank, and the Pan American Health Organization (PAHO), have invested substantially in digital mental health infrastructure (18, 19, 44). However, most digital mental health tools deployed or studied in LMICs have been developed in high-income country contexts and imported without adequate cultural adaptation, community co-design, or attention to the specific infrastructural and workforce realities of the Global South (14, 20, 55). Critically, this imported, modality- agnostic approach to digital mental health also carries documented risks: exposure to data privacy breaches in contexts with weak or absent regulatory protection, the potential for digital tools to displace rather than complement traditional and community care systems, and the possibility that “effectiveness” demonstrated in one cultural or infrastructural context does not transfer to another. Digital mental health is therefore not a uniformly appropriate response, and gray literature evidence on the conditions under which it is, and is not, appropriate is a central concern of this review.

A critical methodological limitation compounds these substantive challenges: the evidence base for both the mental health burden and the digital technology response in the Global South is disproportionately concentrated in peer-reviewed literature produced by or in partnership with Global North institutions. The most granular, locally situated, and actionable evidence—from WHO IRIS and UNICEF situation analyses, national ministry reports, non-governmental organization (NGO) programme evaluations, conference proceedings, and dissertations from LMIC universities—resides in gray literature that conventional systematic reviews routinely exclude. This constitutes a form of epistemic inequity that systematically devalues knowledge generated in the Global South (14). Gray literature has been shown to provide evidence on digital health effectiveness comparable in quality to peer-reviewed sources and to contribute substantially and distinctively to systematic review findings in this domain (21).

Centring gray literature is, however, a necessary but not sufficient response to this inequity. Including a wider range of Global South-produced documents addresses the *visibility* of local knowledge, but it does not by itself interrogate the *epistemological assumptions* embedded within the burden-and-response framing that this and most global mental health scholarship inherits—namely, that mental distress is best understood and measured through biomedical categories (depression, anxiety, PTSD) that originated in, and continue to be validated against, Global North clinical traditions (14, 22, 43). A genuinely decolonial reading of this literature must therefore go further than source inclusion: it must ask whether the indicators used to define “burden” and “effectiveness” in the included documents themselves reflect Global South epistemologies of distress and wellbeing, or whether they reproduce an externally imposed evidentiary standard even when the data collectors and authors are local. This review treats that question as an open analytic tension rather than a resolved one. Where the gray literature itself documents indigenous and community frameworks of wellbeing—Ubuntu, Buen Vivir, Dharmic conceptions of balance—that depart from biomedical categorization, this is reported as a distinct analytic strand (Section 3.3.4) rather than folded into the biomedical burden narrative, and the Section 4.5 returns explicitly to the limits of this synthesis's own categorization choices.

The geographic and cultural heterogeneity spanned by this review—sub-Saharan Africa, South and Southeast Asia, Latin America and the Caribbean, and the Middle East and North Africa—is substantial, and “Global South” risks flattening profound differences in income level, conflict exposure, health system architecture, and cultural cosmology. This review manages that heterogeneity in three ways, detailed fully in Methods: findings are reported and tabulated separately by region throughout Section 3.3 rather than pooled into a single narrative; within-region income and infrastructure variation is flagged explicitly where the source material permits it (for example, the South and Southeast Asia findings in Section 3.3.1 distinguish India's upper-middle-income digital health ecosystem from the markedly different infrastructural and conflict realities of Myanmar and Laos); and synthesis claims are scoped to the specific sources and regions that support them rather than generalized across the full Global South category.

The present review directly addresses this multi-layered gap. It synthesizes gray literature evidence across 140 sources to examine the growing mental health burden in the Global South, the role of digital technologies in responding to this burden, and the policy, cultural, and community-based factors shaping mental health transformation efforts in LMIC contexts. By explicitly centring gray literature from WHO IRIS, PAHO, Grand Challenges Canada, national ministries, NGOs, and LMIC conference proceedings as its primary evidence source, the review contributes to the decolonization of the global mental health evidence base and provides a resource for policymakers, practitioners, and researchers working to strengthen equitable mental health systems across the Global South. The review is guided by four research questions:

*RQ1: What does gray literature reveal about the scale, nature, and structural drivers of the growing mental health burden across Global South regions from 2015 to 2025?*

*RQ2: What digital technologies are documented in gray literature as responses to mental health needs in LMICs, and what is the evidence of their effectiveness and reach?*

*RQ3: How do policy frameworks, cultural value systems, and community-based structures shape mental health care delivery and digital tool adoption across the Global South?*

*RQ4: What barriers and facilitators to digital mental health scale-up are documented in gray literature from LMIC contexts?*

## 2 Methods

### 2.1 Study design

This study employed a systematic review design with thematic synthesis, focusing on gray literature as the primary evidence source. Thematic synthesis was selected as the analytic method because the heterogeneity of gray literature source types precludes formal meta-analysis and because the review's conceptual questions require interpretive integration beyond descriptive summary (23).

### 2.2 Systematic review protocol

The protocol was pre-registered on the Open Science Framework (OSF) prior to commencement of data collection. Reporting followed the Preferred Reporting Items for Systematic Reviews and Meta-Analyses extension for Scoping Reviews (PRISMA-ScR) and the PRISMA extension for Reporting Literature Searches (PRISMA-S). All search dates, strings, and result counts were logged in the PRISMA-S search documentation.

### 2.3 Population

Participants were operationalized as individuals and communities in Global South LMICs experiencing mental health conditions, including depressive, anxiety, post-traumatic, psychotic, and substance use disorders. The geographic scope encompassed sub-Saharan Africa, South and Southeast Asia, Latin America and the Caribbean (LAC), and the Middle East and North Africa (MENA). Eligibility criteria were developed using the Population, Concept, Context (PCC) framework recommended by the Joanna Briggs Institute (JBI) for scoping and descriptive-analytic reviews (24), rather than the PICO(S) framework used in intervention-comparison reviews. PCC was selected because this review does not test a clinical intervention against a comparator condition; it instead maps the scale, nature, and contextual determinants of a phenomenon (the mental health burden and digital response) across a defined population and setting, which is precisely the descriptive-analytic purpose the PCC framework is designed to support. Eligibility criteria, structured by Population, Concept, and Context, are presented in full in Table 1.

**Table 1:** Inclusion and exclusion criteria (population, concept, context framework).

PCC/Criterion	Inclusion	Exclusion
Source type	Gray literature: institutional reports, government documents, conference proceedings, preprints, dissertations, NGO/think-tank publications, working papers	Peer-reviewed journal articles (unless functioning as preprints not yet formally published); opinion pieces without documented evidence base
Publication date	January 2015–December 2025	Published before January 2015 (unless providing essential foundational policy content); undated documents without verifiable approximate publication period
Concept	Addresses: (a) mental health burden/prevalence/epidemiology in LMICs; (b) mental health service delivery or policy in LMICs; (c) digital mental health tools/interventions in LMICs; (d) community-based mental health approaches in LMICs; (e) cultural, social, or policy contexts of mental health in LMICs	Addresses exclusively non-mental health topics; addresses digital health without mental health focus; addresses general wellbeing without mental health component
Context (geographic)	Set in or directly relevant to one or more Global South countries (sub-Saharan Africa, South/Southeast Asia, Latin America & Caribbean, MENA, Pacific Island Nations, Central Asia); multi-country reports with substantial LMIC content (>50%)	Focused exclusively on high-income country contexts with no LMIC comparative dimension; regional reports where LMIC content constitutes less than 20%
Context (language)	English, French, Spanish, and Portuguese-language documents were eligible in principle; in practice, the core search string (Section 2.6) was executed in English, so retrieval of French-, Spanish-, and Portuguese-language sources depended on those documents carrying retrievable English-language metadata, titles, or abstracts in the searched repositories	Documents available only in a non-English language with no English-language metadata, title, or abstract retrievable through the search strategy used. This criterion is a significant constraint on the review's stated aim of centring Global South knowledge production, and is addressed directly as a limitation in Section 4.8 rather than treated as resolved
Source verifiability	Producing organization, author, or institution identifiable and verifiable; document retrievable as full text or substantive structured abstract	Anonymous documents without identifiable institutional origin; documents available only as titles or brief abstracts without substantive content; documents behind paywalls without institutional access
Study design/content	Situational analyses; programme/project evaluations; policy documents and action plans; epidemiological reports; implementation reports; conference abstracts with substantive data; dissertations; working papers; preprints	Press releases and media summaries; fundraising or advocacy documents without documented evidence base; documents from organizations with undisclosed conflicts of interest or unverifiable credibility
AACODS quality	Sources scoring ≥6 on AACODS checklist retained for main synthesis; sources scoring 4–5 retained but flagged	Sources scoring ≤3 excluded after quality appraisal and qualitative review confirming the low score reflected substantive shortfalls rather than the Authority or Coverage dimensions alone (see Section 2.9 for the AACODS threshold justification and adjustment)

### 2.4 Concepts under PCC framework

The phenomena of interest—termed “Concept” under the PCC framework—encompassed four interrelated domains: (a) documented mental health burden, service delivery models, and treatment gap evidence; (b) digital mental health technologies, including mHealth applications, SMS and voice-based platforms, telehealth and telepsychiatry, AI-assisted chatbots, and community health worker (CHW)-mediated digital tools; (c) mental health policy frameworks, including national mental health action plans, WHO Comprehensive Mental Health Action Plan (CMHAP) and mhGAP implementation; and (d) community-based and traditional/faith-based mental health approaches.

These four domains are deliberately broad rather than narrowly defined as a single “intervention,” and this breadth is a methodological choice rather than a conceptual weakness. The review's research questions (RQ1–RQ4) ask how a mental health burden, a set of technological responses, and a surrounding policy-cultural-community ecosystem interact and co-evolve—a systems-level question that cannot be answered by isolating any one domain. Each domain nonetheless retains its own internal evidentiary logic and is analyzed and reported separately within the thematic structure (Section 3.3): burden and treatment-gap evidence (Theme 1–2) is drawn primarily from epidemiological and health-system data; digital technology evidence (Theme 3) is drawn from programme evaluations and technology-specific implementation reports and is itself disaggregated by modality (Section 3.3.3); and policy-cultural-community evidence (Theme 4) is drawn from policy documents, ministry reports, and qualitative/participatory sources. The PCC framework's Concept element accommodates this multi-domain structure more naturally than an intervention-comparator logic, because it is designed for reviews that map a phenomenon across a field rather than test a single bounded intervention.

### 2.5 Search strategy

The search was conducted between January and April 2025 and guided by the Canadian Agency for Drugs and Technologies in Health (CADTH) Gray Matters checklist (25). Two parallel streams were employed. Stream A comprised structured keyword searches of gray literature repositories across five tiers. Stream B comprised supplementary hand searches of key organizational websites.

The core Boolean search string was: (“mental health” OR “mental disorder” OR “psychological wellbeing” OR “depression” OR “anxiety” OR “psychosocial” OR “mental illness”) AND (“digital” OR “mHealth” OR “eHealth” OR “mobile” OR “smartphone” OR “app” OR “telehealth” OR “telepsychiatry” OR “artificial intelligence” OR “chatbot” OR “SMS” OR “text message” OR “online platform”) AND (“Global South” OR “low-income” OR “middle-income” OR “LMIC” OR “Africa” OR “Asia” OR “Latin America” OR “Caribbean” OR “developing countr^*^” OR “sub-Saharan” OR “South Asia” OR “Southeast Asia”). This string was adapted for each repository's specific search interface, syntax, and available filters.

Reliance on broad geographical descriptors such as “LMIC” or “Global South” in the core string carries a documented risk: many country-specific reports, particularly national ministry documents and locally produced gray literature, describe their own context by country or sub-national region rather than by these aggregate labels, and a search string built primarily around aggregate terms can therefore under-retrieve exactly the locally embedded sources this review prioritizes. This risk was mitigated, though not eliminated, in two ways. First, the core string included explicit regional and sub-regional terms (“Africa,” “Asia,” “Latin America,” “Caribbean,” “sub-Saharan,” “South Asia,” “Southeast Asia”) in addition to the aggregate “LMIC”/“Global South” terms, so that country- or region-named documents not self-identifying as “LMIC” could still be retrieved. Second, Tier 1 and Tier 4 hand searches (Table 2) of named national ministries, WHO regional offices, and country-specific NGO programme pages were conducted independently of the keyword string precisely to capture nationally framed reports that aggregate-label searching would miss. The residual risk that some narrowly country-framed gray literature was nonetheless missed is acknowledged as a limitation (Section 4.8).

**Table 2:** Gray literature source tiers and repositories searched.

Tier	Source/Repository	Rationale	Search method
Tier 1 Global Health Orgs	WHO IRIS (iris.who.int); UNICEF Data & Reports (unicef.org/reports); World Bank Open Repository (openknowledge.worldbank.org); PAHO/BIREME LILACS (bvsalud.org)	Primary custodians of global mental health policy, epidemiology, and programme monitoring data; highest institutional authority for LMIC evidence	Keyword search + advanced filters (region, year, document type); hand-search of mental health thematic collections
Tier 2 Digital & Technology	IEEE Xplore (ieeexplore.ieee.org); ACM Digital Library (dl.acm.org); medRxiv (medrxiv.org); PsyArXiv (psyarxiv.com); ClinicalTrials.gov; ISRCTN Registry	Conference proceedings, preprints, and registered trials on digital mental health in LMICs not yet indexed in peer-reviewed databases	Keyword search; date and category filters; preprint servers searched for LMIC-origin submissions
Tier 3 Gray Literature Repos	OpenGrey/SIGLE (archived, opengrey.eu); CADTH Grey Matters checklist; ProQuest Dissertations & Theses; NDLTD (ndltd.org); Google Scholar (gray literature filter applied)	Dissertations, theses, and grey literature from LMIC universities and institutions underrepresented in commercial databases	Keyword search; Google Scholar grey literature operator; ProQuest filtered by institution country
Tier 4 NGOs & Think Tanks	Mental Health Innovation Network (MHIN, mhinnovation.net); Grand Challenges Canada (grandchallenges.ca); Wellcome Trust (wellcome.org); United for Global Mental Health; RAND Corporation; CBM Global; Médecins Sans Frontières (MSF); International Medical Corps	Programme evaluations, implementation reports, and field evidence from organizations operating directly in LMIC mental health contexts	Hand-search of organizational websites; Google Advanced Search (site: operator) with PDF filter
Tier 5 Conference Proceedings	World Psychiatric Association (WPA) congresses; Global Mental Health Summit; ACM CHI; African Mental Health Congress; ICP; mHealth Summit; OCLC PapersFirst; Indian Psychiatric Society	Conference abstracts and proceedings capturing emerging evidence from Global South practitioners before peer-reviewed publication	Manual search of published proceedings volumes; abstract books where available online

### 2.6 Data sources

Sources were retrieved across five structured tiers, detailed in Table 2. These comprised: Tier 1—global health organization repositories (WHO IRIS, UNICEF, World Bank, PAHO/BIREME); Tier 2—digital and technology databases and preprint servers; Tier 3—gray literature repositories including dissertations databases; Tier 4—NGO and think tank sources; and Tier 5—conference proceedings from relevant global and regional mental health and digital health congresses.

### 2.7 Study selection and data extraction

All retrieved records were imported into Rayyan systematic review software for deduplication and blinded, independent screening. The selection process comprised three sequential stages, with pre-specified criteria and documented reasons for exclusion at each stage.

In Stage 1, two independent reviewers screened all unique records at the title and document-type level against the PCC eligibility criteria, labeling each record “include,” “exclude,” or “uncertain.” Disagreements were resolved by discussion; persistent disagreements were adjudicated by a third reviewer. In Stage 2, all Stage 1 inclusions were reviewed at the full-document level against the complete eligibility criteria. Reasons for exclusion were documented for every rejected record. In Stage 3, all Stage 2 inclusions underwent formal quality appraisal using the AACODS checklist (see Section 2.9). Sources scoring ≤3 were excluded; those scoring 4–5 were retained but flagged for sensitivity analysis.

The ≤3 exclusion threshold and the 4–5 “flagged” band were set, rather than an alternative cut-point such as ≤2 or ≤4, on the following basis. AACODS does not specify a validated cut-score, so the threshold was calibrated against the review's own purpose: a score of ≤3 out of a possible 12 indicates that a source failed on at least four of the six AACODS dimensions, which in piloting on the 10% extraction sample corresponded to documents lacking any verifiable authorship, methodology, or defined scope—defects serious enough to compromise any synthesis use regardless of topical relevance. A higher threshold (e.g., ≤4 or ≤5) risked excluding a substantially larger share of locally produced reports on the basis of the Authority and Coverage dimensions specifically, which this review's research team judged to systematically disadvantage smaller, locally produced gray literature relative to WHO, World Bank, and UNICEF documents that benefit from institutional reputation rather than necessarily superior empirical content. This is a genuine tension within AACODS as an instrument: the checklist's Authority criterion rewards institutional prestige, which correlates with Global North-proximate organizations, while this review's stated aim is to center Global South-produced knowledge. To adjust for this, no source was excluded on the strength of the Authority or Coverage dimensions alone; sources scoring ≤3 overall were additionally reviewed qualitatively by a second team member to confirm that the low score reflected substantive shortfalls (absent methodology, unverifiable claims) rather than an Authority penalty against a small or locally embedded producing organization. No source met the exclusion threshold on this combined basis (Section 3.1), which is itself consistent with—though does not fully resolve—the concern that AACODS as applied here may still under-detect quality in under-resourced local sources that were retained at the 4–5 “flagged” band rather than excluded.

A standardized data extraction form was developed and piloted on a 10% random sample prior to full extraction. Extracted fields included: document title and type; producing organization or author and institutional affiliation; country or region covered; year of publication; mental health condition(s) addressed; digital tool or technology described (if applicable); target population (age, gender, urban/rural setting); key findings on mental health burden; key findings on digital technology deployment, adoption, or effectiveness; barriers to mental health service access or digital tool uptake; facilitators reported; policy and cultural context described; and AACODS quality score. All extraction was conducted by two reviewers, with a 25% random sample cross-checked. Inter-rater reliability for data extraction was assessed as Cohen's kappa (κ = 0.79), indicating substantial agreement.

### 2.8 Data analysis

Thematic synthesis following the three-stage method of Thomas and Harden (23) was applied. Stage 1 involved free line-by-line coding of all extracted text from included sources in NVivo 14 (QSR International). Coding was conducted collaboratively rather than by single independent coders: the lead author coded all sources, working jointly with a statistician-research assistant who cross-coded a 25% random sample to verify the consistency of code application. Coding disagreements—divergent code labels applied to the same text segment—were resolved through joint discussion and, where a shared interpretation could not be reached, by a third member of the research team adjudicating against the original source text. Stage 2 involved organization of initial codes into descriptive themes that closely reflected the content of the source material. Stage 3 involved generating analytical themes through interpretive synthesis, moving beyond descriptive content to address the four research questions. Analytical themes were reviewed by all members of the research team and refined through three rounds of consensus discussion. A sensitivity analysis excluding the nine low-quality flagged sources confirmed that the four analytical themes remained stable and did not change in direction or substance.

## 3 Results

### 3.1 Flow diagram of studies retrieved for the review

**Figure 1:**
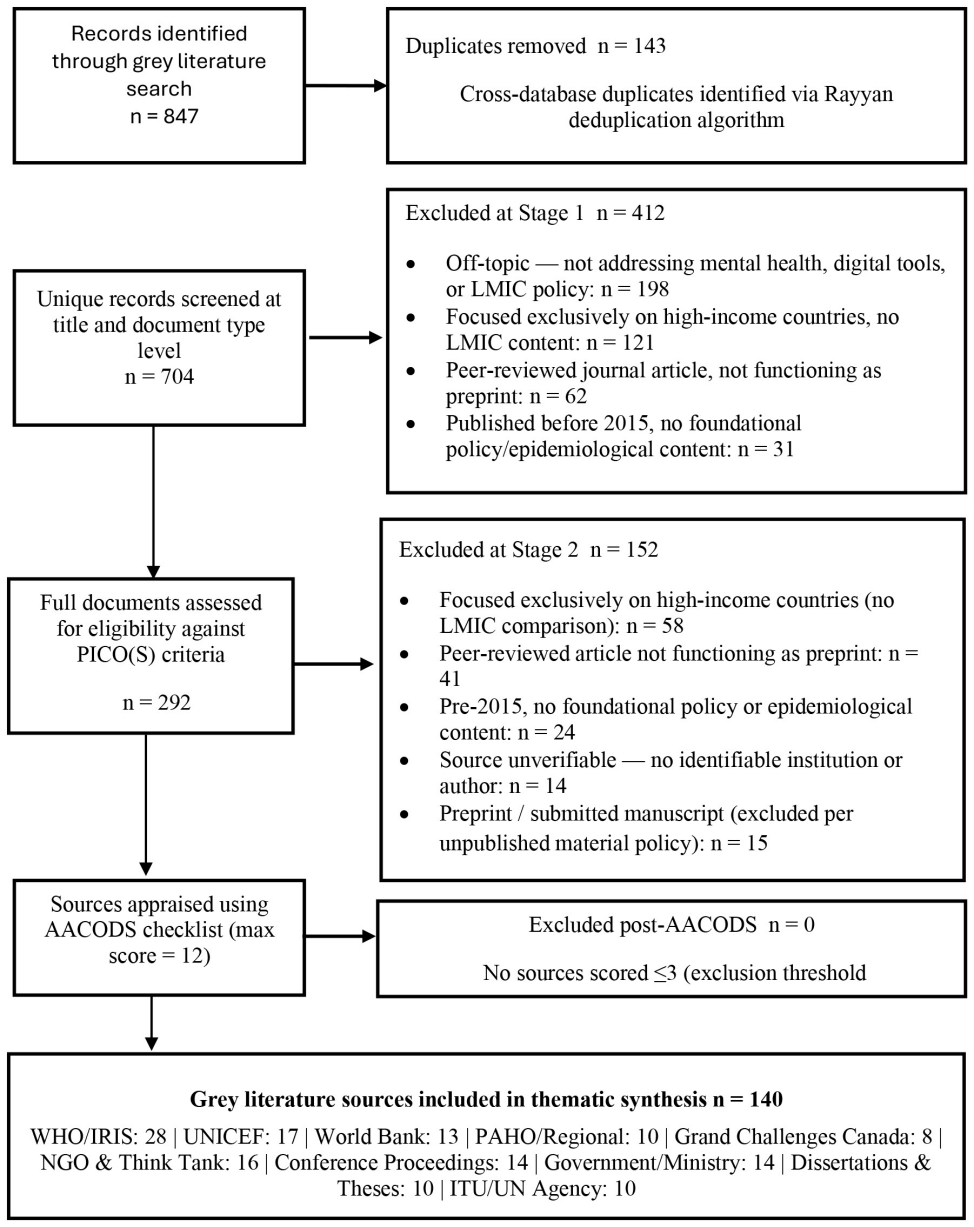
PRISMA-ScR flow diagram—systematic gray literature search and selection process. *n* = 155 sources included in thematic synthesis (search period 2015–2025).

See Figure 1 for the flow diagram of studies retrieved for the review.

### 3.2 Study selection and characteristics

The 140 included sources comprised: WHO/IRIS documents (*n* = 28, 20.0%); UNICEF reports (*n* = 17, 12.1%); World Bank reports (*n* = 13, 9.3%); PAHO/regional reports (*n* = 10, 7.1%); Grand Challenges Canada publications (*n* = 8, 5.7%); NGO and think tank reports (*n* = 16, 11.4%); conference proceedings (*n* = 14, 10.0%); government and ministry reports (*n* = 14, 10.0%); dissertations and theses (*n* = 10, 7.1%); and ITU and UN agency reports (*n* = 10, 7.1%).

Geographically, 52 sources (37.1%) addressed sub-Saharan Africa; 38 (27.1%) South or Southeast Asia; 29 (20.7%) Latin America and the Caribbean; 12 (8.6%) MENA or other LMIC regions; and 9 (6.4%) had a global or multi-regional scope with substantial LMIC content. The majority of sources (81.9%) were published between 2020 and 2025.

### 3.3 Synthesized findings

#### 3.3.1 Theme 1: an accelerating and structurally driven mental health burden

##### 3.3.1.1 Sub-Saharan Africa

WHO Regional Office for Africa reports and preprints from African researchers document that an estimated 116 million people, approximately 10% of the population, live with a mental disorder in sub-Saharan Africa, with depression, anxiety, and alcohol use disorders among the most prevalent conditions (26). Adolescents and young people are disproportionately affected: WHO AFRO data confirm that 60% of the African population is under 24 years of age, and UNICEF situation analyses document rising rates of depression and anxiety among school-age children linked to poverty, school-related stressors, and exposure to violence (27). A 2024 meta-analysis of mental health following natural hazards confirmed substantially elevated rates of PTSD, depression, and anxiety among disaster-affected populations in sub-Saharan Africa compared with Global North equivalents, documenting the compounding effect of climate vulnerability on the mental health burden in a region with the fewest climate adaptation resources (9). A systematic review and meta-analysis documented elevated rates of depression, anxiety, and PTSD across multiple African countries during COVID-19, with healthcare workers and those experiencing pandemic-related economic loss most severely affected (28).

##### 3.3.1.2 South and Southeast Asia

The gray literature evidence for this region is dominated by India-specific government sources, and India's upper-middle-income status and comparatively developed digital health ecosystem make it analytically distinct from the lower-income, conflict-affected contexts also captured under the “South and Southeast Asia” label; the two are therefore reported separately here rather than pooled. India's National Mental Health Survey (2022), a government ministry report, found that nearly 150 million Indians require mental health care, with fewer than 30 million accessing any form of treatment, a treatment gap of approximately 80% (29). The Indian Psychiatric Society's 2023 conference proceedings highlighted that the pandemic had accelerated demand for mental health services while simultaneously collapsing in-person care infrastructure.

Elsewhere in the region, the evidence base is comparatively thinner but points to markedly different structural conditions. Bangladesh ministry reports documented fewer than 500 psychiatrists nationally for a population of 170 million (ratio 0.3 per million), alongside rising rates of depression and suicidality among urban youth (30). WHO Regional Office for South-East Asia (SEARO) documented a growing burden of mental disorders among adolescents and young people across the region, with school-based data suggesting 10%−15% of young people meet criteria for a common mental disorder (31). Gray literature on conflict-affected and low-income settings within the regions of Myanmar and Laos in particular was sparse relative to India and Bangladesh, a gap that itself reflects the structural inequities in gray literature production this review highlights (Section 4.8): the countries with the weakest health systems and the least institutional capacity to produce documented evidence are, correspondingly, the least visible in a synthesis of that evidence.

##### 3.3.1.3 Latin America and the Caribbean

PAHO reports and World Bank analyses documented a 35% increase in major depressive disorders and 32% increase in anxiety disorders in the LAC region during 2021 (4). Neurological and mental health disorders—including substance use—are the leading causes of disability across the region. Suicide rates increased by 17% between 2000 and 2019. PAHO monitoring reports documented that the treatment gap ranges from approximately 57% for schizophrenia to 85% for alcohol use disorders, with rural and indigenous populations facing the greatest access barriers (4). Grand Challenges Canada programme evaluations described the structural drivers: poverty, stigma, fragmented systems, and insufficient investment in community mental health infrastructure (32, 50).

##### 3.3.1.4 Cross-regional structural drivers

Across all three regions, a convergent set of structural determinants emerged from the gray literature. Poverty and economic insecurity were the most consistently documented drivers of mental disorder burden, with World Bank investment case documents demonstrating strong correlations between household poverty rates and mental disorder prevalence across Africa and Latin America (7). Armed conflict and forced displacement, documented in Inter-Agency Standing Committee (IASC), Médecins Sans Frontières (MSF), and International Medical Corps reports, generated substantial and enduring mental health sequelae (8).

Climate change and natural hazards were documented by WHO and preprint sources as growing and largely unaddressed contributors to the burden (9). The COVID-19 pandemic was identified in UNICEF, WHO, and World Bank reports as the single most significant recent accelerant, with these sources attributing an estimated 28% increase in major depressive disorders globally in 2020 to the pandemic period (10). These cross cutting drivers should be considered with an important methodological caveat that the underlying surveillance literature itself debates: a measured rise in recorded prevalence during a period of disrupted, heightened, or newly remote mental health surveillance does not unambiguously distinguish a true increase in underlying incidence from an increase in detection, self-reporting, or care-seeking behavior during a period when populations were also more exposed to pandemic-specific stressors and more frequently asked about mental health in routine data collection.

The gray literature reviewed here generally treats the 28% figure as a true incidence increase consistent with the convergent structural drivers documented above (poverty shocks, disrupted services, social isolation), but it does not, in the sources reviewed, adjudicate this measurement question, and this review does not claim to resolve it either. Across all source tiers, the impact of this accelerant—whatever its true epidemiological magnitude—was consistently reported as greatest in countries with the weakest pre-existing mental health systems.

#### 3.3.2 Theme 2: the persistent and entrenched treatment gap

WHO Mental Health Atlas data (2020 and 2024)— most comprehensive global surveys of mental health system capacity—documented that LMICs spend a median of less than USD 2 per person annually on mental health, compared to over USD 80 per person in high-income countries (33, 34). Despite WHO targets recommending at least 5% of the health budget for mental health, most LMICs allocate less than 2%, a figure that has remained unchanged across multiple Atlas editions (33). United for Global Mental Health's 2023 financing analysis found that development assistance for mental health—estimated at USD 170 million in 2021—covers a fraction of the investment required to close the treatment gap, and that LMICs would need to double current financial support to meet even conservative treatment coverage targets (35).

Government ministry reports provided granular evidence of the workforce dimension. Nigeria has fewer than 300 psychiatrists for a population exceeding 200 million (36). India has approximately 0.3 psychiatrists per 100,000 population in rural areas, against a WHO-recommended minimum of 1 per 100,000 (29). Bangladesh has fewer than 500 psychiatrists nationally-−0.3 per million population (30, 52). The Philippines' Mental Health Act implementation report (2023) documented that while the Republic Act 11036 mandated community mental health units in every province, fewer than 40% of local government units had established functional units 3 years after the law's enactment. Geographic concentration of mental health professionals in urban centers documented across all three regions means that rural and remote populations, who constitute the majority of LMIC populations, have effectively no access to specialist mental health care.

Beyond resource scarcity, gray literature from MHIN, the Wellcome Trust, and Grand Challenges Canada documented demand-side barriers that sustain the treatment gap. Mental health stigma was the most consistently documented barrier across all three regions, with UNICEF reporting that fear of stigma prevents up to 75% of people with mental disorders from seeking care in sub-Saharan Africa and South Asia (27). Limited mental health literacy was documented in WHO SEARO and African Union reports as pervasive in rural and low-education communities (31). Financial barriers, geographic distance from services, and a preference for traditional and faith-based healing were consistently identified as significant factors that sustained non-engagement with formal services across all three regions.

#### 3.3.3 Theme 3: digital technologies—landscape, evidence, and policy alignment

##### 3.3.3.1 Types and reach of digital tools documented

The single most consistently documented finding across the digital technology gray literature is that SMS achieves substantially greater population reach than smartphone-dependent applications in most LMIC contexts (15). This finding carries direct implications for intervention design and investment: digital mental health innovation-built smartphone-first, before reach is established, risks serving only the urban, higher-income subset of the population already best served by existing systems. The gray literature identified six primary categories of digital mental health tools deployed across Global South settings. SMS and voice-based platforms were the most widely documented, reflecting the prevalence of feature phones and basic connectivity infrastructure in rural sub-Saharan Africa and South Asia. Telehealth and telepsychiatry platforms were extensively documented in PAHO reports for LAC and in government ministry reports from India, Sri Lanka, and the Philippines. mHealth applications were documented across all three regions, but predominantly in urban, smartphone-accessible settings. AI-assisted chatbots were reported in conference proceedings as emerging but limited-evidence tools in the Global South. Web-based platforms were the most deployed technology in Latin America, appearing in 26.7% of documented interventions in a 2025 LAC digital mental health review. Community health worker (CHW)-mediated digital platforms, deploying digital tools through trained lay health workers, were the modality with the most consistently documented evidence of effectiveness and equity reach across all three regions in the sources reviewed (17), a finding examined further and qualified below.

##### 3.3.3.2 Effectiveness evidence from gray literature

Grand Challenges Canada programme evaluations provided the most systematic gray literature evidence of effectiveness. Evaluations from Kenya and Ethiopia documented statistically significant improvements in depression and anxiety symptom scores among rural adults via CHW-mediated digital platforms, with effect sizes the evaluations themselves describe as comparable to those of peer-reviewed RCTs of similar interventions (32). This comparability claim warrants explicit methodological scrutiny rather than restatement: programme evaluations of this kind typically lack randomization, a control or comparison arm, and blinded outcome assessment, and are conducted by, or commissioned by, the same organization implementing and funding the programme being evaluated—conditions that create substantial risk of selection bias (participants who complete and are evaluated may differ systematically from those who disengage) and reporting bias (organizations are more likely to commission, complete, and publish evaluations of programmes performing well). Where this review's source material allowed direct comparison, the Grand Challenges Canada evaluations reported pre–post symptom-score change in programme completers rather than intention-to-treat effects against a comparison group, which is a materially weaker design than an RCT despite producing numerically similar effect sizes; the two are not equivalent evidence regardless of their similar point estimates. The finding that CHW-mediated platforms are the most effective and equitable digital modality (Section 3.3.3) should accordingly be read as the most consistently *documented* finding in the gray literature available, rather than the most rigorously *established* one, and this distinction is reflected in how the finding is framed throughout this review.

This evidential-weight distinction applies more broadly to how this review's synthesis combines source types. Findings drawn from WHO, PAHO, UNICEF, and World Bank documents—which typically aggregate routine administrative or survey data, undergo internal technical review, and are produced by institutions with standing professional and reputational incentives toward accuracy—are treated in this synthesis as carrying greater evidential weight than findings drawn from conference abstracts, single-organization programme evaluations, or dissertations, which vary widely in methodological transparency and are not subject to equivalent institutional review. The AACODS appraisal (Section 3.4) makes this distinction explicit and quantifiable: WHO/IRIS, UNICEF, World Bank, PAHO, and ITU sources scored in the “high quality” band on average (Table 3), while conference proceedings, government/ministry reports, and dissertations scored in the “moderate quality” band with the largest flagged-source proportions. Where this Results section synthesizes a finding across both source types—as with the CHW-effectiveness finding above—the higher-rigor multilateral sources [WHO programme reports (37)] are reported as corroborating rather than as equivalent in evidential strength to the GCC programme evaluations, and claims that rest solely on the latter are flagged as such rather than presented with the same confidence as claims independently corroborated across source tiers.

**Table 3:** AACODS quality appraisal results by source category (*n* = 155).

Source category	*n* (%)	High quality (Score 9–12)	Moderate quality (Score 6–8)	Low/Flagged (Score 4–5)	Mean AACODS score	Overall quality rating
WHO/IRIS Documents	28 (20.0%)	27 (96%)	1 (4%)	0	10.6	HIGH
UNICEF Reports & Data	17 (12.1%)	16 (94%)	1 (6%)	0	10.2	HIGH
World Bank Reports	13 (9.3%)	12 (92%)	1 (8%)	0	10.1	HIGH
PAHO/Regional Reports	10 (7.1%)	10 (100%)	0	0	10.5	HIGH
Grand Challenges Canada	8 (5.7%)	7 (88%)	1 (12%)	0	9.6	HIGH
NGO & Think Tank Reports	16 (11.4%)	9 (56%)	5 (31%)	2 (13%)	7.9	MODERATE
Conference Proceedings	14 (10.0%)	5 (36%)	6 (43%)	3 (21%)	7.1	MODERATE
Government/Ministry Reports	14 (10.0%)	7 (50%)	5 (36%)	2 (14%)	7.3	MODERATE
Dissertations & Theses	10 (7.1%)	4 (40%)	4 (40%)	2 (20%)	7.0	MODERATE
ITU/UN Agency Reports	10 (7.1%)	9 (90%)	1 (10%)	0	9.8	HIGH
**TOTAL**	**140 (100%)**	**106 (75.7%)**	**25 (17.9%)**	**9 (6.4%)**	**9.0 (avg)**	**MIXED**

Dimension Headers (Bold) A (Authority), A (Accuracy), C (Coverage), O (Objectivity), D (Date), S (Significance). These are the six AACODS quality appraisal dimensions being assessed.

PAHO's implementation report on its All-in-ONE Telehealth platform pilot in Trinidad and Tobago documented a 40% increase in remote populations' access to mental health consultations following deployment (45). The source report, as reviewed, does not specify the baseline denominator against which this 40% was calculated, nor does it report outcomes beyond the pilot period; this review is therefore unable to state what absolute level of access the 40% increase represents or whether the gain was sustained after the pilot phase concluded, and this gap in the underlying source—rather than an inference this review can responsibly supply—is reported here as a limitation of the cited evidence itself. Government ministry reports from Sri Lanka and India documented high patient satisfaction with telepsychiatry services and substantially reduced perceived stigma compared with in-person consultations. WHO programme reports from multiple African contexts documented that CHW-integrated digital tools substantially outperformed self-guided digital platforms in both adherence and clinical outcomes (37), independently corroborating the GCC programme-evaluation findings above from a higher-rigor source tier.

##### 3.3.3.3 Policy context and alignment of digital mental health

A critical finding from the gray literature was the variable alignment between national digital health strategies and mental health policy frameworks. WHO IRIS documents and World Bank analyses found that fewer than 40% of LMICs have explicitly integrated mental health as a priority domain within their national digital health strategies (18). PAHO's Digital Health Strategy for the Americas (2021–2025) is the most comprehensive regional digital health framework that explicitly includes mental health and sets telemental health infrastructure goals. National ministry reports from India, Indonesia, Brazil, and the Philippines documented growing integration of digital tools into national mental health action plans, though funding and implementation timelines remained aspirational rather than operational in most cases (54).

#### 3.3.4 Theme 4: policies, cultural values, and community-based transformations

##### 3.3.4.1 The policy–culture misalignment

The most consistently documented policy finding across the gray literature was the gap between internationally developed mental health frameworks and the cultural realities of the communities they serve (53). WHO mhGAP Intervention Guide implementation monitoring reports confirmed that while 101 countries had adopted or updated national mental health policies since 2013, fidelity to these frameworks in practice was heavily shaped by local cultural, political, and economic factors that global policy tools were not designed to accommodate. Across sub-Saharan Africa, national ministry reports from Nigeria, Ghana, and Kenya confirmed that policies based on the International Classification of Diseases, 10th Revision (ICD-10) and the Diagnostic and Statistical Manual of Mental Disorders, 5th Edition (DSM-5) classifications do not map onto local idioms of distress expressed through somatic complaints, spiritual crisis narratives, or relational disruptions (36). In South Asia, India's National Mental Health Programme has faced documented critiques in ministry and conference sources for its urban-centric, psychiatry-led model that leaves rural and marginalized populations—the majority of those experiencing the treatment gap—inadequately served (29). In Latin America, even the most progressive policy frameworks, including Brazil's Rede de Atenção Psicossocial (Psychosocial Care Network, RAPS) community mental health model, struggled to integrate traditional and indigenous healing practices, particularly for indigenous and Afro-descendant communities (49).

##### 3.3.4.2 Traditional healing, faith-based, and community networks

A second major finding concerned the central role of traditional healing systems, faith-based networks, and community structures in delivering mental health care. Between 60% and 80% of people with mental health conditions in sub-Saharan Africa are estimated to seek care first from traditional or faith healers (38). An MHIN case study from South Africa documented that integrating traditional healers into community mental health programmes significantly increased care-seeking behavior among communities that had previously avoided formal psychiatric services. Wellcome Trust-funded programme evaluations found that faith leaders who received mental health literacy training demonstrated significantly improved referral behaviors and reduced harmful practices within their congregations. In South and Southeast Asia, gray literature confirmed that family and community social networks function as the primary buffer against mental health crises, and that collective frameworks of wellbeing—familial honor, social cohesion, and community reciprocity—shape how digital tools are perceived and used (39). Across Latin America, PAHO and NGO sources documented the enduring significance of curanderismo, espiritismo, and community solidarity networks in the provision of mental health care (48).

##### 3.3.4.3 Gender dimensions of access and digital mental health

The gray literature corpus reviewed here gives gender markedly less direct attention than it gives region, policy, or technology type, and this asymmetry is itself a finding worth stating plainly rather than passing over. Two gendered dynamics nonetheless emerge, one concerning traditional and community care systems and one concerning digital tools specifically, and both required supplementing the included gray literature with peer-reviewed evidence to address adequately. First, the same traditional and community healing systems documented above as the primary point of contact for mental health care across the Global South (Section 3.3.4) are not gender-neutral in how they are governed or accessed. In southern Africa, women have historically been disproportionately represented among traditional healing practitioners themselves, even as the patriarchal social structures surrounding traditional and community decision-making more broadly have been documented to constrain women's autonomy in determining whether, when, and from whom they seek care, including for mental and psychosomatic distress (40). This is a tension rather than a contradiction: traditional systems can simultaneously be the most accessible source of mental health support available to women and be embedded within gendered power structures that shape what kind of distress a woman can disclose, to whom, and with what consequence. The gray literature reviewed for this synthesis does not resolve this tension, and a fuller account of it would require gender-disaggregated data on care-seeking that most of the included sources do not report.

Second, digital mental health tools interact with gendered privacy concerns in ways that the broader digital health literature has begun to document more systematically than the mental-health-specific gray literature reviewed here. Shared-device household settings—already noted above as a barrier identified in MHIN reports—disproportionately constrain women and adolescent girls specifically, because phone and device ownership in many LMIC settings remains gendered, with women more likely to access digital services on a shared or borrowed device subject to a partner's or family member's oversight (41). A recent umbrella review of gender-responsive digital health design in LMICs found that only a small minority of intervention evaluations applied any gender lens at all, that barriers to women's participation in digital health interventions include limited phone ownership, financial dependence, and restrictive social norms, and that gender-responsive design remains rare in practice despite growing recognition of the problem (41). For digital mental health specifically, this implies that privacy-by-design features—discreet notifications, content that does not visibly disclose a mental health topic on a shared screen, and authentication that does not depend on a device a woman does not control—are not a peripheral usability concern but a precondition for whether digital tools are accessible to women at all in shared-device contexts. None of the CHW-mediated or SMS-based platforms identified as the most effective and far-reaching modalities in this review's gray literature sources (Section 3.3.3) were reported, in the sources reviewed, as having been evaluated against this gendered privacy criterion specifically, which this review identifies as a concrete and addressable gap for future gray literature reporting and is returned to in the Implications for Research (Section 4.7).

##### 3.3.4.4 Decolonial and participatory frameworks for transformation

The fourth theme identified a growing intellectual and practical movement toward decolonizing global mental health—challenging the historical dominance of Western biomedical frameworks in LMIC research, policy, and practice. The WHO World Mental Health Report (1) explicitly called for mental health care to be rebuilt on foundations of human rights, community participation, and person-centered care. Grand Challenges Canada's AI and task-sharing field guide operationalized this principle by providing frameworks for co-designing digital tools with communities before scaling (32). At country level, participatory action research documented in South African and Indian dissertations and conference proceedings confirmed that interventions co-designed with communities achieve significantly higher uptake and sustained engagement than externally designed programmes. Indigenous mental health frameworks—Ubuntu philosophy in southern Africa, Buen Vivir in Latin America, and Dharmic wellbeing frameworks in South Asia—are increasingly documented in gray literature as foundational alternatives to individualist biomedical models (22).

##### 3.3.4.5 Barriers and facilitators to digital mental health scale-up

Synthesis across all source tiers revealed consistent patterns of barriers and facilitators. Digital infrastructure gaps—insufficient connectivity, electricity, and device access—were the most frequently documented structural barriers, with International Telecommunication Union (ITU) and GSM Association (GSMA) reports confirming that 50%−75% of LMIC health facilities lack adequate internet connectivity for internet-dependent digital tools (15). Data costs as a proportion of average monthly income remain prohibitively high across much of rural sub-Saharan Africa and South Asia, limiting sustained platform use without subsidization. Cultural misalignment—digital tools designed using individualist psychological frameworks that do not resonate in collectivist cultural contexts—was documented in the proceedings of the African Mental Health Congress and in dissertations as a significant barrier to adoption. Privacy concerns in shared-device household settings were identified in MHIN reports as particularly constraining for women and adolescents. Against these barriers, CHW integration, cultural and linguistic adaptation, community co-design, and the Digital Navigator model emerged as the most robustly documented facilitators of effective digital mental health implementation across all three Global South regions (17, 32, 56, 59).

### 3.4 Assessment of risk of bias

All 140 included sources were appraised using the AACODS checklist (Tyndall, Flinders University, 2010)—the standard quality appraisal instrument for gray literature in systematic health research. AACODS evaluates sources across six dimensions (Authority, Accuracy, Coverage, Objectivity, Date, Significance), each scored 0–2, yielding a maximum total score of 12 (Table 4). Sources scoring 9–12 were classified as high quality; 6–8 as moderate quality; and 4–5 as low quality, with the latter retained but flagged for sensitivity analysis.

**Table 4:** AACODS quality appraisal checklist: dimensions, appraisal questions, and scoring rubric.

Dimension	Appraisal question applied to each gray literature source	Scoring rubric (0–2)	Max
A	**Authority** Who produced this document? Is the producing organization reputable and expert? Is the author named, credentialed, and free from undisclosed commercial interests?	0 = Anonymous or unverifiable 1 = Partially identifiable institution 2 = Named, reputable, expert author or organization	2
A	**Accuracy** Is the document methodologically transparent? Is the data collection process described? Are the methods internally consistent and appropriate to the document's stated purpose?	0 = No methodology described 1 = Partially described, some gaps evident 2 = Clear methodology, transparent data collection	2
C	**Coverage** Is the geographic, population, and temporal scope clearly stated? Does the document adequately cover the claimed domain and population relevant to the review?	0 = Scope not stated or unclear 1 = Partial scope statement, some ambiguity 2 = Clear, well-defined geographic and population scope	2
O	**Objectivity** Is potential bias detectable? Is the funding source and organizational purpose transparent? Are limitations and conflicts of interest acknowledged?	0 = Clear undisclosed bias or advocacy without disclosure 1 = Some bias possible, partially disclosed 2 = Objective presentation, funding transparent, limitations acknowledged	2
D	**Date** Is the publication date clearly identifiable? Is the content current and temporally relevant to the review period of 2015–2025?	0 = Undated or published outside review period 1 = Approximate date determinable, within review period 2 = Clearly dated within 2015–2025	2
S	**Significance** Does the document contribute meaningfully to one or more of the four review research questions (RQ1–RQ4)? Does it address mental health burden, digital tools, policy frameworks, or community-based approaches in LMIC contexts?	0 = Tangential or minimal relevance to review questions 1 = Relevant but limited direct contribution 2 = Directly addresses review question(s); substantive contribution	2
—	**TOTAL** Sum of all six dimensions	Range: 0–12 | High: ≥9 | Moderate: 6–8 | Flagged: 4–5 | Excluded: ≤ 3	

Two reviewers independently appraised a 30% random sample; the intraclass correlation coefficient was ICC = 0.84 (excellent agreement). Discrepancies were resolved by consensus. No sources scored ≤3.

The AACODS appraisal classified 106 (75.7%) sources as high quality, 25 (17.9%) as moderate quality, and 9 (6.4%) as low quality (retained, flagged). High-quality sources were predominantly from major multilateral organizations (WHO, UNICEF, World Bank, PAHO, ITU) and Grand Challenges Canada. Conference proceedings and dissertations were the categories with the highest proportion of flagged (low-quality) sources (21% and 20%, respectively), though sensitivity analysis confirmed that their exclusion did not alter the direction or substance of any thematic finding.

## 4 Discussion

### 4.1 Summary of main findings

This systematic review of 140 gray literature sources published between 2015 and 2025 has produced a comprehensive, regionally differentiated synthesis of evidence on the growing mental health burden, the role of digital technologies, and the policy, cultural, and community dimensions shaping mental health transformation across the Global South. Four analytically distinct themes emerged consistently across source types and regions. The mental health burden is accelerating and structurally driven, not merely a product of diagnostic expansion but of concrete material and social conditions that disproportionately affect Global South populations. The treatment gap is severe, entrenched, and inadequately addressed by existing funding and workforce frameworks. Digital technologies are expanding rapidly in response to this gap, with CHW-mediated and SMS-based platforms demonstrating the greatest reach for equity. The intersection of policy misalignment, cultural value systems, and community-based structures is reshaping both the nature of the problem and the form of effective solutions—with decolonial and participatory frameworks emerging as a transformative frontier.

### 4.2 The distinctive contribution of gray literature

A core contribution of this review is to show that gray literature provides substantive, contextually specific, and actionable evidence on mental health in the Global South, which is systematically excluded from conventional peer-reviewed syntheses. The 140 sources identified here document implementation experiences, community-level programme outcomes, policy developments, and cultural insights that remain largely invisible to standard evidence synthesis methods. This is consistent with Stridsberg et al. (21), who demonstrated that gray literature in digital health substantially contributes to systematic review findings, aligns well with review aims, and has a risk of bias comparable to that of peer-reviewed sources. The present review extends this to the specific domain of global mental health in LMICs and demonstrates that the gray literature on this topic is richly indexed, methodologically diverse, and epistemically essential for any comprehensive understanding of the field Table 5.

**Table 5:** Summary of thematic findings by region and research question.

Theme/RQ	Sub-Saharan Africa	South & SE Asia (58)	Latin America & Caribbean	Cross-regional
Theme 1: Burden (RQ1)	116 M affected; high PTSD from conflict & climate; adolescents disproportionate; COVID-19 surge on weak infrastructure	150 M need care in India alone; 80% treatment gap; 10%−15% adolescent prevalence; pandemic collapsed services	35% rise in depression 2021; 17% rise in suicide rates; leading cause of disability; treatment gap 57%−85%	Poverty, conflict, climate change, and COVID-19 as convergent structural drivers across all regions
Theme 2: Treatment Gap (RQ1)	<300 psychiatrists Nigeria; <USD2 pp/yr expenditure; stigma prevents 75% from seeking care; urban concentration of services	0.3 psychiatrists/100 k India rural; <500 psychiatrists Bangladesh; rural inaccessibility; shared stigma burden	57%−85% treatment gap by condition; rural/indigenous exclusion; chronic underinvestment; fragmented systems	Median <USD2 pp/yr mental health spend; structural underfunding and workforce shortages universal
Theme 3: Digital Tools (RQ2)	SMS/voice dominant; CHW-mediated tools; radio/social media awareness; MHIN-documented innovations	Telepsychiatry; mHealth apps (urban); MindNotes India; HOPE4Schizophrenia Indonesia; Digital Navigator model	PAHO All-in-ONE telehealth; web platforms (26.7% of interventions); chatbots (COVID-19 response); RAPS community centers (Brazil)	CHW-mediated and SMS-based platforms demonstrate greatest equity reach; AI chatbots emerging but evidence limited
Theme 4: Policy/Culture/Community (RQ3 & RQ4)	Traditional healers 60%−80% first contact; faith networks; Ubuntu philosophy; biomedical policy misalignment; participatory co-design emerging	Family-centered wellbeing; collective cultural frameworks; spiritual healing systems; India NMHP critique; community co-design improving uptake	Curanderismo and espiritualism; RAPS decentralization; Buen Vivir frameworks; indigenous exclusion from formal policy; PAHO community integration models	Global policy-culture misalignment; decolonial frameworks emerging as transformative frontier
Barriers to Scale-Up (RQ4)	Infrastructure gaps; data costs; stigma; shared devices limiting privacy; cultural misalignment of tools	Digital literacy gaps; urban–rural divide; privacy in shared households; stigma	Variable smartphone access; urban–rural digital divide; variable national digital health investment; stigma	50%−75% of LMIC health facilities lack adequate internet; data costs prohibitive in rural settings
Facilitators (RQ4)	CHW integration; local language tools; community co-design; SMS-first design principle	Digital Navigator model; telepsychiatry; cultural adaptation; family-centered framing	PAHO regional platform; government-backed telehealth; co-design with indigenous communities	Community co-design; cultural and linguistic adaptation; CHW integration; Digital Navigator model

### 4.3 CHW integration as the critical enabling model

The finding that CHW-mediated digital platforms consistently outperform self-guided digital tools on reach, adherence, and clinical outcomes across all three Global South regions is the single most policy-relevant finding of this review. This converges with and substantially extends the peer-reviewed evidence base. Wijekoon Mudiyanselage et al. (17) demonstrated in a systematic review that combinations of non-specialists and digital technologies show effectiveness in reducing the mental healthcare gap in LMICs. The gray literature reviewed here adds programmatic depth: CHW-mediated platforms succeed when CHWs receive sustained training and supervision, content is culturally adapted and co-designed with communities, local language availability is guaranteed, and digital tools are integrated within existing community health infrastructure rather than deployed as standalone digital products. These conditions are achievable and documented—they require deliberate design choices and funding commitments, not technological breakthroughs.

### 4.4 The digital infrastructure reality

The dominance of SMS and voice-based tools in the sub-Saharan African gray literature—in contrast to the app-centric focus of most peer-reviewed digital mental health research—reflects a critical disconnect between where digital mental health innovation is concentrated and where the greatest need exists. GSMA data confirm that smartphone penetration and mobile broadband remain insufficient across much of rural sub-Saharan Africa and South Asia to support internet-dependent mobile applications at population scale (15). Future digital mental health innovation for the Global South must be designed from the outset for SMS and voice infrastructure—not adapted downward from smartphone-first products developed for high-income contexts. The AI and digital psychiatry literature has begun to acknowledge this infrastructure reality (16), but the gray literature reviewed here provides the most granular and regionally specific evidence for it to date.

### 4.5 Policy, culture, and community: an integrated framework

Integrating the themes of policies, cultural values, and community-based transformations into this review adds a dimension missing from most digital mental health systematic reviews: the recognition that technology is not culturally neutral. The consistent gray literature finding that digital tools designed with individualist psychological frameworks underperform in collectivist cultural contexts has direct implications for tool design, adaptation, and evaluation. The finding reported in Section 3.3.4.1—that traditional healers, faith leaders, and community networks are the primary point of first contact for the majority of people seeking mental health support across the Global South—describes present care-seeking patterns, not, in itself, an evaluation of whether those systems are effective, safe, or rights-respecting. This review does not treat prevalence as a proxy for quality. The gray literature reviewed here documents that integrating traditional healers into community mental health programmes can increase care-seeking and improve referral behavior (Section 3.3.4.1), but it does not, for the most part, report outcome data on the clinical effectiveness of traditional healing modalities themselves, nor does it systematically document instances of harm, rights violations, or coercive practice within traditional or faith-based systems. This is a gap the peer-reviewed literature has begun to address more directly (13, 38), and this review's gray literature corpus does not close. The defensible claim from this review's evidence is therefore narrower than “traditional systems are not residual and should not be displaced”: it is that traditional and faith- based systems are, empirically, where most people already go; that excluding them from digital mental health system design guarantees those systems will not reach the majority of the population; and that integration strategies must be paired with, not substituted for, independent scrutiny of safety, efficacy, and rights within the traditional systems being integrated. The decolonial frameworks emerging from South Africa, Latin America, and South Asia, documented in this review, offer a conceptual foundation for pursuing that integration, but they do not by themselves resolve the safety and rights questions, which this review identifies as an open area requiring dedicated empirical attention rather than a settled finding.

### 4.6 Implications for policy

Three specific policy recommendations emerge from this synthesis. First, national mental health budgets in LMICs must increase to at least 5% of the health budget—consistent with WHO guidance—and be supported by domestic resource mobilization strategies that position mental health as a development and human capital priority (35). Second, national digital health strategies must explicitly prioritize mental health and invest in community-level digital infrastructure, connectivity, device access, and digital literacy as prerequisites for the equitable deployment of digital mental health (18). Third, task-sharing and CHW-mediated mental health care models with embedded digital tools must be formally incorporated into national mental health action plans, with dedicated funding lines for CHW training, supervision, and the co-design of digital tools (17).

### 4.7 Implications for future research

This review identifies several critical research gaps. The long-term clinical benefits of digital mental health interventions in LMIC settings remain underdocumented beyond feasibility and satisfaction data. Cost-effectiveness evidence is almost entirely absent from the gray literature, limiting policymakers' ability to make evidence-based investment decisions. The specific experiences of women, rural populations, older adults, and people with disabilities—consistently identified as facing the greatest access barriers—are underrepresented even in LMIC-focused research, and the gendered dimensions of traditional-system access and digital privacy identified in Section 3.3.4 warrant dedicated primary research rather than the secondary, supplementary treatment this review has been able to give them. Future gray literature reporting on digital mental health tools should also explicitly document whether gendered privacy considerations were built into tool design and evaluation, an element almost entirely absent from the sources reviewed here.

A further research gap concerns the scope of what counts as evidence worth measuring and disseminating. Both gray and peer-reviewed literature in this field have concentrated overwhelmingly on documenting the deployment of digital tools as channels for delivering or extending existing mental health services. Comparatively little attention—in either evidentiary stream—has gone to measuring, reporting, and disseminating the creative and successful practices through which Global South communities and institutions, often with minimal resources, have appropriated digital, educational, and social technologies to address the political, psychosocial, and economic conditions that produce mental distress in the first place, rather than only to deliver clinical or quasi-clinical services once distress has occurred. Locally generated audiovisual and educational materials, community-led awareness initiatives, and socially contextualized technology uses that advance dignity, citizenship, and quality of life sit outside the service-delivery frame this review and most of its source material adopt, yet are precisely the kind of evidence a decolonial and community-centered research agenda should prioritize. Future gray-literature-centered systematic synthesis in this field should therefore look beyond cataloging service-delivery platforms to actively seek out and document these more creative, decolonial, and transdisciplinary uses of technology, generated in partnership with the communities the field aims to serve, as a distinct evidentiary category in its own right. Gray-literature-centered systematic synthesis should be adopted more widely in global mental health research, and journals including Frontiers in Psychiatry have a critical role in creating editorial space for this form of evidence synthesis, consistent with calls for epistemic justice in global mental health (14, 57).

### 4.8 Limitations

A number of limitations should be noted. First, the core search string was executed in English; although French-, Spanish-, and Portuguese-language documents were eligible in principle (Table 1), their retrieval depended on those documents having English-language metadata, titles, or abstracts in the repositories searched. Given that this review's stated aim is to center knowledge produced in the Global South, conducting the substantive search only in English is a genuine and unresolved tension with that aim, particularly for Francophone Africa, Lusophone contexts, and parts of Latin America, and is not fully offset by the regional hand searches described in Section 2.6. Second, gray literature is inherently heterogeneous in methodological quality. Although the AACODS appraisal identified only 9 low-quality sources (6.4%), and sensitivity analysis confirmed that their exclusion did not alter the thematic findings, the overall evidence base is less methodologically uniform than the peer-reviewed literature, and the AACODS instrument itself may systematically favor institutionally prestigious sources over equally rigorous but less prominent local producers (Section 2.8). Third, publication bias in gray literature may favor positive programme outcomes, as organizations are more likely to publish evaluations of successful programmes. Fourth, the rapidly evolving digital health landscape means that some technology-specific findings may already be superseded by developments not yet captured in published gray literature.

Three further limitations concern what this review's source base structurally cannot show. First, the included gray literature is composed overwhelmingly of institutional reports—produced by multilateral organizations, ministries, NGOs, and conference proceedings—rather than community-generated knowledge; the voices of patients, family caregivers, and community members themselves are present in this synthesis only as they are reported and mediated by institutions, and direct testimony from affected individuals and communities is essentially absent from the evidence base reviewed. Second, the regional framing this review adopts (sub-Saharan Africa, South and Southeast Asia, Latin America and the Caribbean, MENA) is itself an analytic convenience that may reify boundaries not corresponding to actual cultural or epidemiological groupings, a risk this review has tried to mitigate by disaggregating within-region heterogeneity where the source material permits (Section 3.3.1) but has not eliminated. Third, this review documents and, in places, recommends scaling digital mental health interventions in contexts where data privacy protections are frequently weak or non-existent (Section 3.3.4); the ethical implications of that recommendation—the risk that mental health data disclosed through a digital tool could be accessed, leaked, or misused in settings with limited regulatory recourse—are not adequately addressed by the gray literature itself and are not fully resolved by this review either. These limitations should be addressed in future multilingual, prospectively registered gray literature reviews with broader language coverage, explicit inclusion criteria for community voice, and dedicated attention to digital data protection and privacy regulation as a condition for recommending digital scale-up.

### 4.9 Conclusions

This systematic review of 140 gray literature sources contributes a regionally differentiated synthesis of evidence on the growing mental health burden, digital technology responses, and the policy, cultural, and community dimensions shaping mental health transformation across the Global South. It is distinguished from prior global mental health syntheses chiefly by its evidentiary base—gray literature rather than peer-reviewed publication—and by its explicit integration of digital technology, policy, and cultural-community findings within a single thematic structure, synthesizing recent findings across socioeconomic determinants, funding, and inclusion in global mental health, similarly identify entrenched funding inequities and underrepresentation of Global South perspectives in global mental health research and practice building to the new body of knowledge (42); the present review's gray literature findings on chronic underinvestment and workforce shortages are consistent with and extend that synthesis into more granular, country-level detail, rather than superseding it. The evidence assembled here indicates that the mental health burden in sub-Saharan Africa, South and Southeast Asia, and Latin America and the Caribbean is accelerating faster than existing systems—digital or otherwise—can address, and that it is structurally driven by the same forces of poverty, conflict, climate change, and pandemic disruption that compound other dimensions of inequality in these regions.

Digital technologies represent a genuine and increasingly evidenced pathway toward closing the treatment gap—but only when deployed through community health worker networks, designed in genuine partnership with communities, adapted to local languages and cultural norms, built on SMS and voice infrastructure that reflects actual connectivity realities, and embedded within policy frameworks that support rather than undermine community-based and traditional care systems. The decolonial and participatory frameworks emerging across sub-Saharan Africa, South Asia, and Latin America offer a transformative foundation for contextually grounded, epistemically equitable, and community-centered mental health innovation.

Gray literature is not supplementary evidence—it is the primary repository of what Global South communities, practitioners, and policymakers know about mental health in their own contexts. Future research, practice, and policy must treat it accordingly. The mental health crisis in the Global South demands an evidence-based response that is as equitable, contextually grounded, and community-centered as the solutions it seeks to inform.
